# Synthesis of Self-Healing Waterborne Polyurethane Systems Chain Extended with Chitosan

**DOI:** 10.3390/polym11030503

**Published:** 2019-03-15

**Authors:** Dae-Il Lee, Seung-Hyun Kim, Dai-Soo Lee

**Affiliations:** Division of Semiconductor and Chemical Engineering, Chonbuk National University, Baekjedaero 567, Deokjin-gu, Jeonju, Chonbuk 54896, Korea; ldi4084@naver.com (D.-I.L.); thdnlsla@naver.com (S.-H.K.)

**Keywords:** waterborne polyurethane, self-healing, chitosan, exchange reactions

## Abstract

In this study, the self-healing properties of waterborne polyurethane (WPU) were implemented by chitosan as a chain extender of polyurethane prepolymers. The physical properties and self-healing efficiency of WPU were studied by changing the molar fractions of chitosan from 0.1 to 0.3. After thermal treatment for 24 h at 110 °C, the self-healing efficiency for the tensile strength of the highest chitosan content (WPU-C3) was found to be 47%. The surface scratch was also completely restored. The efficiency of the sample with the lowest chitosan content (WPU-C1) was found to be 35%, while that of the control sample without chitosan (WPU-C0) was 4%. The self-healing properties of the as-prepared films were attributed to the exchange reactions between the hydroxyl groups of chitosan and the urethane groups in the films at elevated temperature. It is inferred that self-healing WPU can be synthesized by chain extension with chitosan.

## 1. Introduction

Recently, self-healing polymers have attracted the attention of many researchers because of their sustainably durable properties. Typically, the technology of self-healing allows for the repair of physical damage and cracks, prolonging the life of a material, particularly those of polymers. In the past several years, efforts have been made to impart polymers with self-healing features, including silicones [1], thermosetting polymers [2,3,4,5], rubbers [6], polyurethanes (PUs) [7,8], and hydrogels [9,10]. Among them, PU has become an attractive candidate with its specific chemical structure and various design flexibilities which have been found to be beneficial for overcoming problems related to the weakness and decreased functionality. The self-healing of PUs can be implemented by their intrinsic and extrinsic properties. Intrinsic self-healing properties of the damage are based on the inherent dynamic nature of physical interactions or covalent bonds in polymers while extrinsic self-healing properties allow them to repair themselves via intentionally pre-embedded healing agents [11]. Many researchers have made great attempts to obtain self-healing PU through the introduction of various reversible chemistries, such as Diels-Alder reaction adducts [12], disulfide groups [8,13,14], hydroxyl groups, imine groups, dynamic urea [15,16], metal ligands [17], and additives [18,19,20]. The exchange reactions using hydroxyl groups for the self-healing PUs have normally been carried out at elevated temperatures using esters, urea, and urethane groups [21]. Testing PUs at room temperatures also have the advantage of exhibiting improved mechanical properties through hydrogen bonding [22].

Waterborne polyurethanes (WPU) were specifically developed to replace solvent based PUs, used for coatings and adhesives, to prevent environmental pollution from organic volatile compounds (VOCs). With the demand for manufacturing WPU systems that are more reliable and sustainable, self-healing WPUs were studied by many researchers inspired by living organisms and tissues. For example, functionalized WPUs have been reported to recover scratches through self-healing [23,24]. However, the performance of WPU films, such as water resistivity, are generally more inferior to those of solvent based PU films due to the introduction of functional groups for the dispersibility of PU in aqueous systems [25].

It is worth mentioning that the chitosan for the modification of waterborne PUs can be obtained from chitin, which is plentiful in nature. According to Fu and coworkers, nanocomposites of waterborne PUs and halloysite modified with chitosan showed a vast improvement in the tensile properties of PU films after drying [26]. Xu et al. reported that the incorporation of chitosan moieties into waterborne PU resulted in better blood compatibility for PU films. The hydrophilicity of PU films can also diminish when chitosan moieties are integrated into the films [27]. Studies of self-healing PUs containing chitosan and oxetane groups have also been reported [28].

**Scheme 1:**
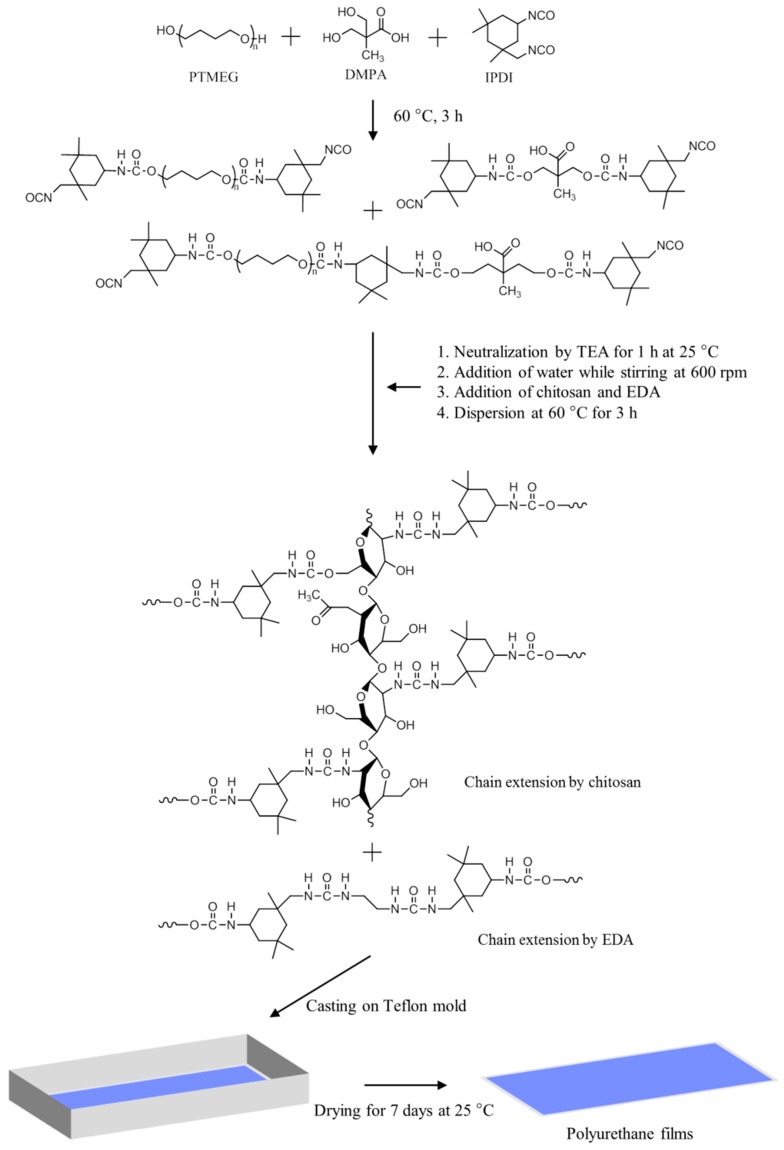
The preparation of waterborne polyurethane (WPU) chain extended with chitosan and ethylenediamine (EDA).

In this study, we use chitosan as a partial chain extender for WPU systems by forming urea groups in the reaction between amine and isocyanate terminated PU prepolymers, as shown in Scheme 1. Among the functional groups available in chitosan, such as amines, hydroxyl and acetate groups, amines are the most reactive groups and react well with isocyanates. Here, the urea will be formed during polymerization. We note that the hydroxyl groups of chitosan in the WPU can undergo an exchange reaction readily with urethane and urea groups at 110 °С, and thus, self-healing WPU films can be obtained. In this paper, self-healing of WPU containing chitosan as a chain extender will be discussed.

## 2. Experimental Section

### 2.1. Materials

Before use, poly(tetramethylene ether glycol) (PTMEG, M_n_ = 2000 g mol^−1^; Sigma-Aldrich, Yong-In, Korea) was dried at 60 °С under vacuum for 24 h. 2,2-Bis(hydroxymethyl) propionic acid (DMPA, Sigma-Aldrich, Yong-In, Korea), isophorone diisocyanate (IPDI, Daejung Chemical, Si-Heung, Korea), triethylamine (TEA, Daejung Chemical, Si-Heung, Korea), ethylenediamine (EDA, Daejung Chemical, Si-Heung, Korea), chitosan (M_n_ = 5000 g mol^−1^, 80% deacetylated; Sigma-Aldrich, Yong-In, Korea), glucosamine (GCM, Daejung Chemical, Si-Heung, Korea), butanol (BOH, Daejung Chemical, Si-Heung, Korea), octylamine (ONH_2_, Daejung Chemical, Si-Heung, Korea), phenyl isocyanate (PI, Kanto Chemical, Tokyo, Japan), and dimethyl acetamide (DMAc) were all used as-received, without purification.

### 2.2. Preparation of Waterborne Polyurethane (WPU)

Stoichiometric amounts of PTEMG, DMPA, and IPDI given in Table 1 were added into a four-necked flask and the reaction mixture was stirred at 60 °С to synthesize –N=C=O (isocyanate) terminated PU prepolymers as shown in the 1st step of Scheme 1. Isocyanate contents of PU prepolymer were determined following ASTM D1638-74 during the syntheses. When the isocyanate content reached the theoretically calculated value for the PU prepolymers after about 3 h, chain extensions were carried out following the 2nd step of Scheme 1. After cooling to room temperature, the required amount of TEA was then added to neutralize the DMPA. Emulsification was accomplished by adding deionized water to get an aqueous PU of 20 wt. % prepolymer at a rotor speed of 600 rpm, to which EDA and chitosan (see Table 1 for molar ratio) were added dropwise to the prepolymer emulsion. After stirring for 3 h at 60 °С, the peaks (~2259 cm^−1^) for –N=C=O groups in FT-IR spectra of samples collected from the emulsions which disappeared as shown in Figure S1 and the polymerization of the WPU was completed. When the chitosan ratio was above 0.4, the WPUs were found to aggregate due to the gelling and instability of the colloidal system. After completion of the reaction, a stable dispersion was obtained. WPU samples became milky with the increasing concentration of chitosan among the chain extenders due to the increase of the particle size as shown in Figure S2. The particle size distribution was measured (Figure S3) and summarized (Table S1). The number average particle size of WPUs increased with increasing the chitosan content due to the increase of gel fraction as shown in Table S2. WPU films were obtained through WPU castings from a Teflon mold. To achieve the desired film, this mold was placed into a convection oven at 25 °С for 7 days to avoid bubbles due to air-trap during the drying process. Scheme 1 illustrates this process. Table 1 summarizes the sample codes and recipes for the WPUs.

### 2.3. Characterization

Fourier transform-infrared (FT-IR) spectra of the as-prepared samples were acquired using an FT/IR 300E (JASCO, Tokyo, Japan) in attenuated total reflection mode. The self-healing properties of the samples were observed using an AIS2100 scanning electron microscope (SEM; Seron Technologies Inc., Gyeonggi-do, Korea). WPU films of 5 mm × 5 mm were fixed with a carbon tape on the plates for scratch-healing test. A thin scratch was made on the surface of WPU film using a razor blade. The surface of the film was coated with gold using an ion coater (HC-21, Hoyeontech, Seongnam, Korea). SEM images of the films were taken after the heat treatment of the scratched films in a convection oven at 110 °С as the repair of scratch at 100 °С was negligible in a preliminary test. Differential scanning calorimetry (DSC) was performed with a Q20 analyzer (TA Instruments Inc., New Castle, DE, USA) at temperatures between −100 and 200 °С, and a heating rate of 10 °С/min. The mechanical properties and cut-and-healing of the as-prepared samples were characterized using a universal materials testing machine (UTM, LR5K plus, LLOYD Instruments, West Sussex, UK). Specimens for the cut-and-healing tests were prepared by cutting the center of dog bone specimens for tensile tests with a razor blade and putting the surface of cut specimens as close as possible and placed in an oven for the self-healing at 110 °C for 24 h. Tensile properties of the specimens were measured at 25 °С with a speed of 500 mm/min after the heat treatment for the self-healing. Three specimens per sample were measured to get the average value. The liquid chromatography-mass spectrometry (LC/MS) spectra for the exchange reactions were obtained using an AGILENT 1100 (Agilent Technologies, Palo Alto, CA, USA). Particle size and distribution were measured by dynamic light scattering spectrometer (UPA-150, Microtrac, Montgomeryville, PA, USA) at room temperature with WPU dispersed in water. The dynamic mechanical properties of the samples were investigated using dynamic mechanical analyzer (DMA, Q800, TA Instruments Inc., New Castle, DE, USA). The sample size was 13 mm × 5 mm × 1 mm (length × width × thickness) and measured from −100 °С to 200 °С at a rate of 5 °С/min. The ^1^H NMR was investigated by 600 MHz FT-NMR spectrometer (JNM-ECA600, JEOL Ltd., Tokyo, Japan). All samples were dissolved in dimethyl sulfoxide d_6_ (DMSO-d_6_) and measured. Small angle X-ray scattering (SAXS) measurements were performed to observe microphase separation properties by D8 Discover (Bruker, Billerica, MA, USA). Here the sample code ‘WPU-Cn treated’ is given to each film of WPU that was heat treated to 110 °С for 24 h.

## 3. Results and Discussion

**Figure 1:**
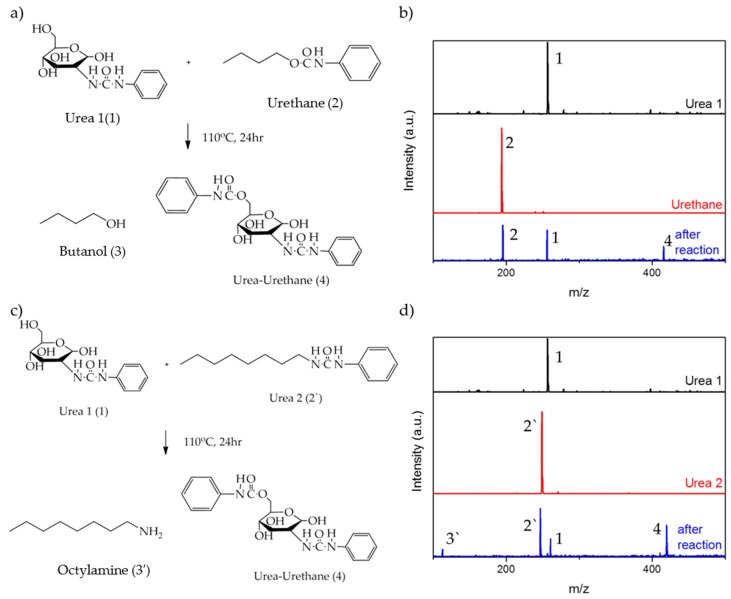
Exchange reactions for model compounds of chitosan, urethane units, and urea units in WPUs: (**a**) a schematic exchange reaction for the hydroxyl groups of chitosan and urethane in WPUs; (**b**) an LC/MS spectrum confirming the scheme introduced in (**a**); (**c**) a scheme of the hydroxyl group exchange reactions in chitosan and urea in WPUs; (**d**) an LC/MS spectrum confirming the scheme introduced in (**c**).

In order to confirm the exchange reactions, model compounds were synthesized and exchange reactions between the model compounds were investigated. The exchange reactions between hydroxyl groups and urethane or urea groups were undertaken at elevated temperatures as shown in Figure 1. The model compound chosen for chitosan moieties in WPUs, Urea 1, was obtained through a reaction among glucosamine and phenyl isocyanate. Model compounds of urethane and urea units in WPUs, Urethane and Urea 2, were also synthesized through a reaction between butanol or octyl amine, and phenyl isocyanate respectively. To confirm the hydroxyl group exchange reaction in Urea 1, Urethane or Urea 2, the mixtures were maintained at a temperature of 110 °C for 24 h. The reactions were confirmed by LC/MS and ^1^H NMR spectra and given in (b) and (d) of Figure 1 and Figures S4 and S5 respectively. In LC/MS data, the intensities of Urea 1, Urethane, and Urea 2 were found to decrease due to the products that were formed from the exchange reaction after treatment at 110 °C. For units containing a hydroxyl group, butanol (3), the molecular weight was found to be so small that it was unobservable when measured with LC/MS. However, in ^1^H NMR spectra given in Figures S4 and S5, all of the products formed by the exchange reactions were observed. With the model compounds, the hydroxyl groups of chitosan were confirmed to have undergone the exchange reaction with urethane groups and urea units.

**Figure 2:**
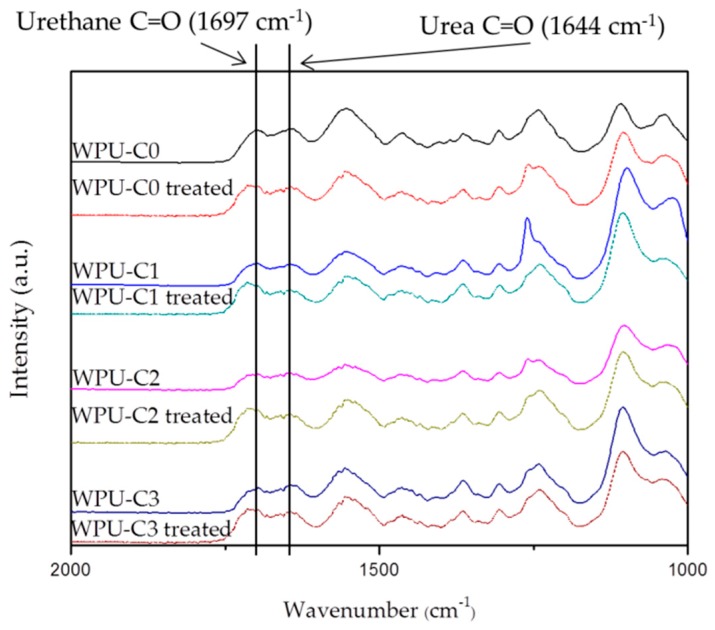
The FT-IR spectra of PU films before and after heat treatment for 24 h at 110 °С.

Figure 2 and Figure S6 give the FT-IR spectra of waterborne polyurethane films prepared at different chitosan concentrations. The characteristic peaks of waterborne polyurethane were found to have appeared at 3320 cm^−1^ for hydroxyl groups (Figure S6) and 1697 cm^−1^ and 1644 cm^−1^ for the carbonyl groups of urethane and urea (Figure 2), respectively. After the heat treatment for 24 h at 110 °С, a change in the intensity ratio of the carbonyl groups of urethane and urea was clearly observed and summarized in Table 2. Compared to the intensity ratio of the carbonyl groups in WPU-C0 and WPU-C0 treated, the intensity ratios in the WPU-C2 and WPU-C3 samples were observed to increase after heat treatment due to the exchange reactions between the hydroxyl groups and urea units as discussed in Figure 1a–c for the model compounds. In addition, an increase in the free carbonyl group of 1714 cm^−1^ can be observed in Figure S7. It can be confirmed that hydrogen bonds are much weakened through the exchange reactions introducing irregularity in the hard segment domains.

**Figure 3:**
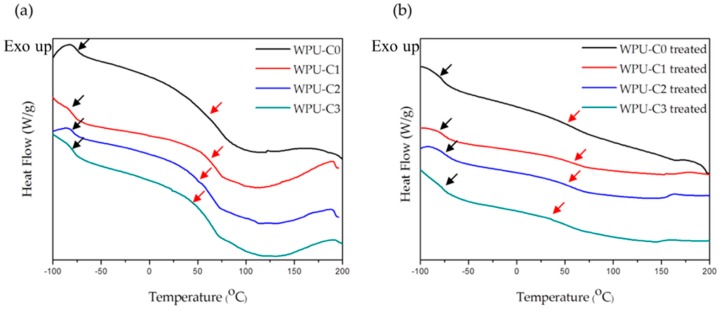
Differential scanning calorimetry (DSC) thermograms for waterborne polyurethane (WPU) films after drying (**a**) and after heat treatment for 24 h at 110 °С (**b**).

**Figure 4:**
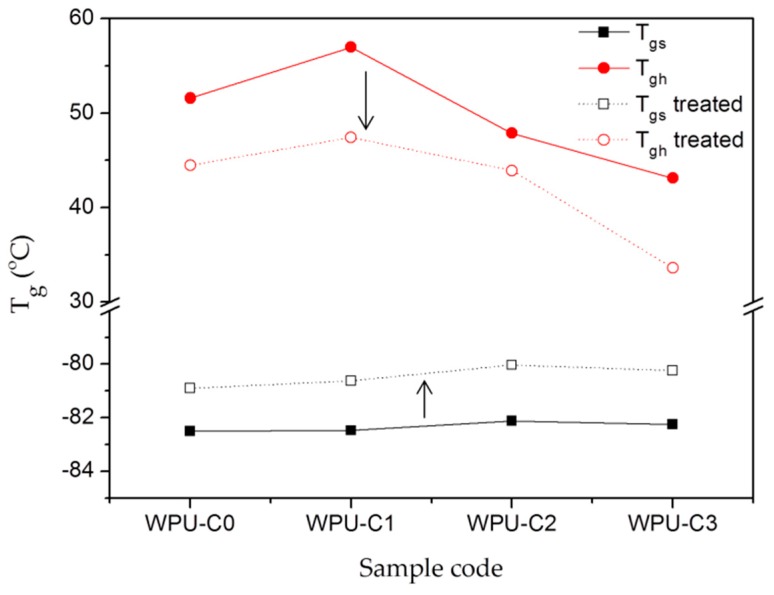
The thermal properties of different waterborne polyurethanes (WPUs) before (solid line) and after (broken line) heat treatment for 24 h at 110 °С.

Figure 3 gives the DSC thermograms of the WPU films prepared using different chitosan concentrations. Glass transition temperatures of soft segments (T_gs_) around −82 °C and hard segments (T_gh_) above room temperature were indicated by arrows in the DSC thermograms. Wide endothermic peaks were observed between 70–150 °C in WPU-C1, WPU-C2, and WPU-C3 after drying. Scanning after the heat treatment yielded reduced endotherms. According to Kittur et al., some bound water could not be removed completely in chitosan systems [29]. The wide endotherms observed in WPU-C1, WPU-C2, and WPU-C3 was attributable to bound water of chitosan moieties. Here, Figure 4 shows the change in glass transition temperature for the WPU films. It can be seen that the T_gh_ increased and, then, experienced a decrease due to a change in microphase separation between the soft segments and the hard segments. Small angle X-ray scattering (SAXS) data reflecting the microphase separations are given in Figure S8. It was found that the inter-domain distance of WPU films increased with increasing the concentration of chitosan due to the different microphase separations. Note that the T_gh_ lowered after each heat treatment. As shown in Figure S9 and Table S3, WPU-C0 and WPU-C1 showed no significant change after heat treatment, but WPU-C2 and WPU-C3 showed the decrease of d-spacing values due to the phase mixing by exchange reactions at the interphase. Conversely, the T_gs_ increased as chitosan was incorporated as a chain extender, and furthermore, the T_gs_ increased after each heat treatment. It was speculated that microphase separation in the WPU films was impeded by the chitosan due to its oligomeric nature, which is unfavorable for hard segment association. Heat treatment to achieve an exchange reaction and self-healing may have also played roles that hinder the microphase separation in the WPU films due to randomized urethane and urea units. This can be seen in Figure 1 for each model system.

**Figure 5:**
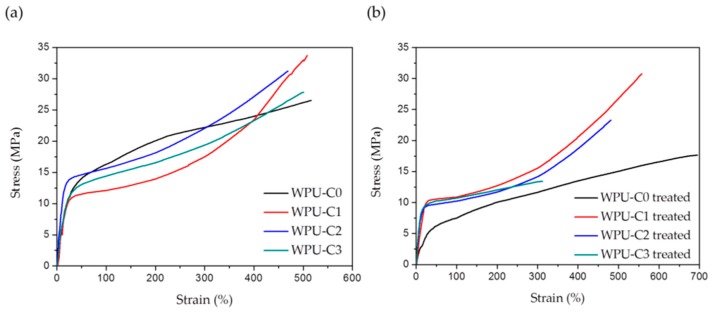
The strain-stress curves for different WPUs: (**a**) samples before heat treatment; (**b**) samples after heat treatment for 24 h at 110 °С.

**Figure 6:**
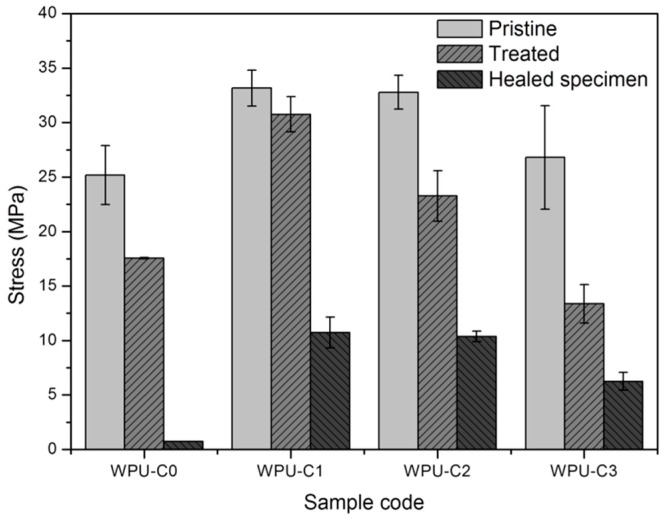
The mechanical properties of different WPUs after the cut-and-heal tests by the heat treatments of pristine specimens.

Figure 5 and Figure 6 show the stress–strain curves obtained from the tensile tests of WPU films. In Figure 5a, it can be seen that the tensile strength of WPU films increased steadily, but then decreased with an increase in chitosan concentration. For WPU-C1, in particular, an ultimate tensile strength of 33.6 MPa was achieved, which was attributed to cross linking by the chitosan chain extension. A further increase in chitosan content, however, only resulted in a decrease in tensile strength due to a decrease of the microphase separation as discussed above in Figure 4 [30,31,32]. Here, WPU films also showed a reduction in tensile properties after heat treating to 110 °C for 24 h (Figure 5b). The changes in tensile properties shown in Figure 5 were attributed to a change in microphase separation through the exchange reaction as discussed in Figure 4 together with Figure S9 and Table S3. Thermal degradation of polyurethanes occurs around 220 °C in general. Thus, the self-healing temperature in this study is far below the thermal degradation temperatures of PU films. Figure 6 shows the results of the heat treatment along with the cut-and heals tests.

The self-healing efficiency of the WPUs was measured through tensile tests using the cut-and-heel method. The film was first cut into a dog bone shape and then cut completely using a razor blade. The cut faces were contacted precisely and healed at 110 °C for 24 h. The tensile properties were then measured and compared with the heat-treated samples. As shown in Table 3, the efficiency of the original WPU was 4%, which is considered to be very low. Conversely, when incorporating a 0.1 molar fraction of chitosan as a chain extender, the efficiency was found to increase to 35%. As the chitosan ratio increased from 0.2 to 0.3, the efficiency increased to 45% and 47%, respectively. This implies that the exchange reaction has been successfully carried out through the hydroxyl groups present in chitosan and that the self-healing process is activated in the PU film of WPU, despite the increase of crosslinking by chitosan. The increase in crosslinking by chitosan was confirmed by measuring the gel content (Table S2).

**Figure 7:**
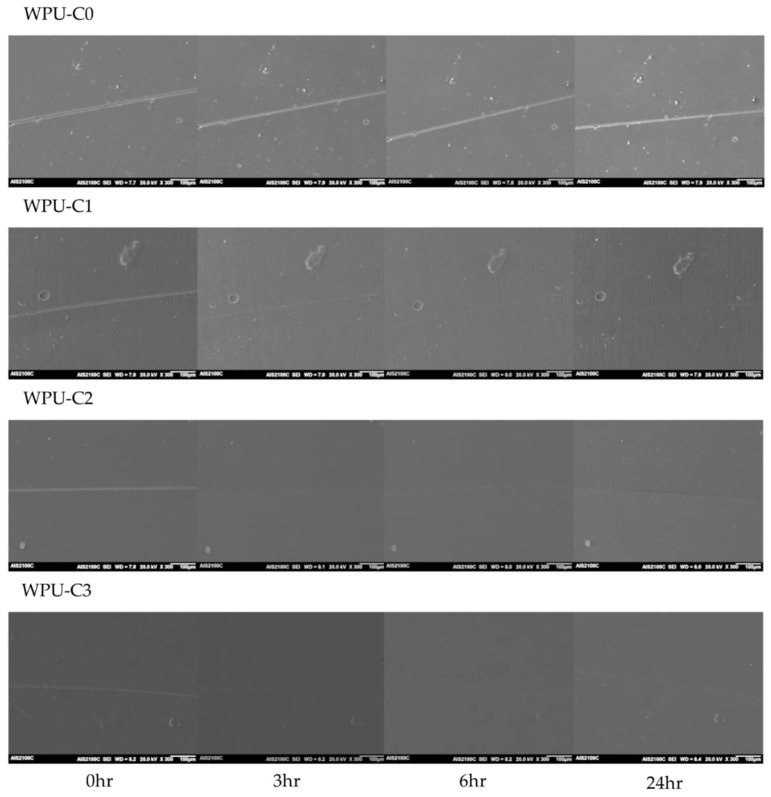
SEM images of the WPU films during the scratch healing process at 110 °С.

To observe the self-healing effects in this study, a scratch healing test was conducted. Here, the WPU film was scratched using a razor blade, with the healing effects observed at 110 °С using SEM. As shown in Figure 7, we confirm that each scratch stayed in place after 24 h for the WPU without chitosan. However, in the WPU films containing chitosan, such scratches disappeared over time. We also found that as the content of chitosan increased, the self-healing effects became more prominent. For example, with WPU-C3 (having the highest amount of chitosan), the scratches disappeared after 6 h. We believe this to be due to the to the hydroxyl groups of chitosan, which undergo an exchange reaction with urea and urethane units in the PU films. In order to confirm the repeated self-healing capabilities, a scratch healing test was conducted with the specimens after the heat treatments and SEM images of the WPU films as shown in Figure S10. Self-healing properties of WPU-C1, WPU-C2, and WPU-C3 were observed also in the specimens heat treated. It is concluded that self-healing WPU can be synthesized by chain extension with chitosan. However, further studies on the progress of self-healing in terms of temperatures and times in WPU films are necessary.

## 4. Conclusions

In this study, we show that when chitosan was added as a chain extender in WPU, the hydroxyl groups of chitosan were able to induce exchange reactions, leading to the self-healing properties of WPU films. The hydroxyl groups present in chitosan appeared to have caused exchange reactions through the nucleophilic addition of urea and urethane groups in the PU at elevated temperatures. The peak intensity rations of urea units and urethane units found in the FT-IR spectra, along with the T_gs_ and T_gh_ of the WPU films were shown to vary due to the exchange reaction and a decreased microphase separation in the PUs. When the chitosan concentration was relatively low (i.e., 0.1 molar fraction), the tensile properties were seen to improve through a slight crosslinking. The resulting films showed good heat stability, but low healing efficiency overall. A further increase in chitosan content resulted in a decrease in microphase separation due to heavy crosslinking in addition to the exchange reactions. This resulted in slightly lower mechanical strength, but a higher healing efficiency.

## Figures and Tables

**Table 1:** Sample codes and recipes for different WPUs.

Sample Code	Composition (by wt. %)					Chain Extender Ratio (in Mols)	
	DMPA	PTMEG	IPDI	EDA	Chitosan	EDA	Chitosan
WPU-C0	6.00	57.02	32.57	4.40	0.00	1.0	0.0
WPU-C1	6.00	55.05	32.13	3.90	2.89	0.9	0.1
WPU-C2	6.00	53.15	31.71	3.43	5.70	0.8	0.2
WPU-C3	6.00	51.29	31.30	2.96	8.44	0.7	0.3

**Table 2:** The absorbance ratio of the urethane (C=O peak) to urea (C=O peak) in an untreated and treated sample of WPU.

Sample Code	Urethane C=O (1697 cm^−1^)/Urea C=O (1644 cm^−1^)	% Increase after Heating
WPU-C0	0.97	
WPU-C0-treated	1.00	3
WPU-C1	1.05	
WPU-C1-treated	1.13	8
WPU-C2	0.93	
WPU-C2-treated	1.11	19
WPU-C3	0.89	
WPU-C3-treated	1.04	17

**Table 3:** The self-healing efficiency of waterborne polyurethanes containing chitosan.

Sample Code	Stress (MPa)		Efficiency (%)
	Before Healing	After Healing	
WPU-C0	17.56	0.74	4
WPU-C1	30.75	10.73	35
WPU-C2	23.26	10.37	45
WPU-C3	13.37	6.25	47

## Supplementary Materials

The following are available online at https://www.mdpi.com/2073-4360/11/3/503/s1, Figure S1: FT-IR spectra of WPU during the chain extension, Figure S2: Images of WPU emulsions, Figure S3: Particle size distribution of WPU emulsion, Figure S4: ^1^H NMR spectra of the model compounds for the exchange reaction Urea-1 and Urethane: (a) butanol; (b) Urethane; (c) products of the exchange reactions between Urea-1 and Urethane, Figure S5: ^1^H NMR spectra of the model compounds for the exchange reaction Urea-1 and Urea-2: (a) octyl amine; (b) Urea-2; (c) products of the exchange reactions between Urea-1 and Urea-2, Figure S6: The FT-IR spectra of PU films before and after heat treatment for 24 h at 110 °С, Figure S7: The FT-IR spectra of PU films before and after heat treatment for 24 h at 110 °С in the wavenumber of 2000~1000 cm^−1^, Figure S8: SAXS data of WPU films before and after heat treatment for 24 h at 110 °С, Figure S9: SAXS data of WPU films before and after heat treatment for 24 h at 110 °С in each sample, Figure S10: SEM images of the WPU films after the second self-healing test by the additional scratch and heat treatments at 110 °С for, Table S1: Number average particle size of WPU emulsions, Table S2: Gel contents of WPU films, Table S3: d-spacing of WPU films based on SAXS data.
