# Supplementary material for: Synthesis of Self-Healing Waterborne Polyurethane Systems Chain Extended with Chitosan
Source: Polymers (Basel). 2019 Mar 15;11(3):503. doi: 10.3390/polym11030503 (PMC6473447; doi:10.3390/polym11030503)
Supplement: Supplementary file 1 [file polymers-11-00503-s001.pdf]

SUPPORTING INFORMATION

Synthesis of Self-Healing Waterborne Polyurethane Systems Chain Extended with Chitosan.

Dae-Il Lee†, Seung-Hyun Kim†, and Dai-Soo Lee*

Division of Semiconductor and Chemical Engineering, Chonbuk National University, Baekjedaero 567, Deokjin-gu, Jeonju, Chonbuk, 54896, Korea; ldi4084@naver.com(DIL); thdnlsla@naver.com(SHK)

† Contributed equally to this work

* Correspondence: daisoolee@jbnu.ac.kr; Tel.: +82-63-270-2310

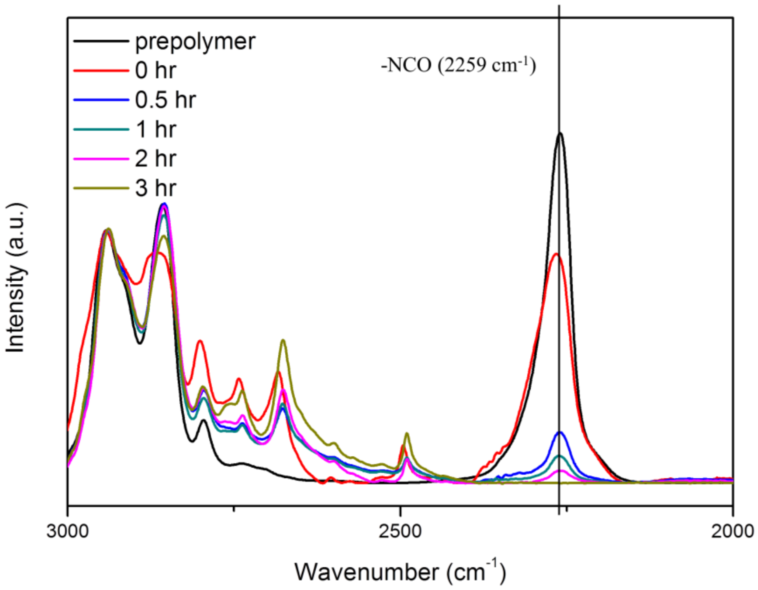

Figure 1. FT-IR spectra of waterborne polyurethanes (WPU) during the chain extension. Peaks due to isocyanate groups at 2259 cm^{-1} disappeared after 3 h.

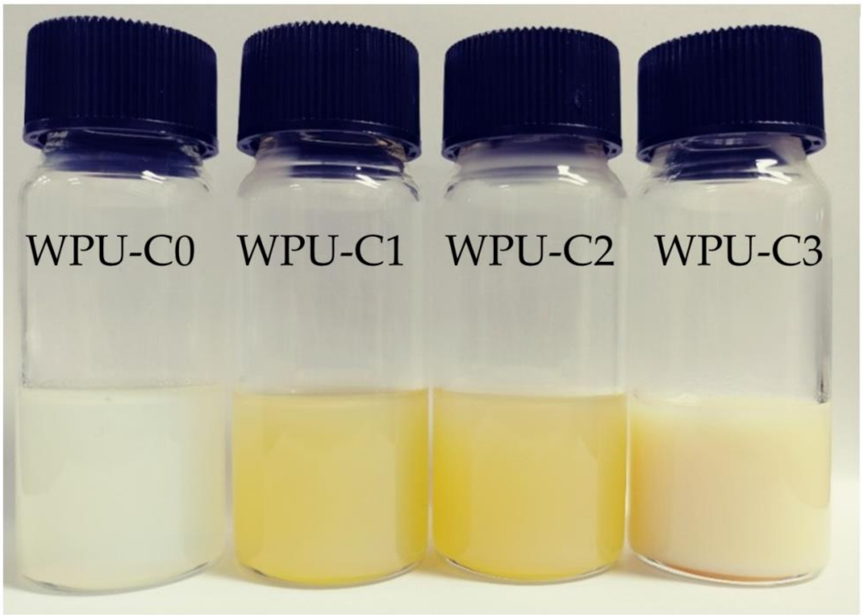

Figure S2. Images of WPU emulsions.

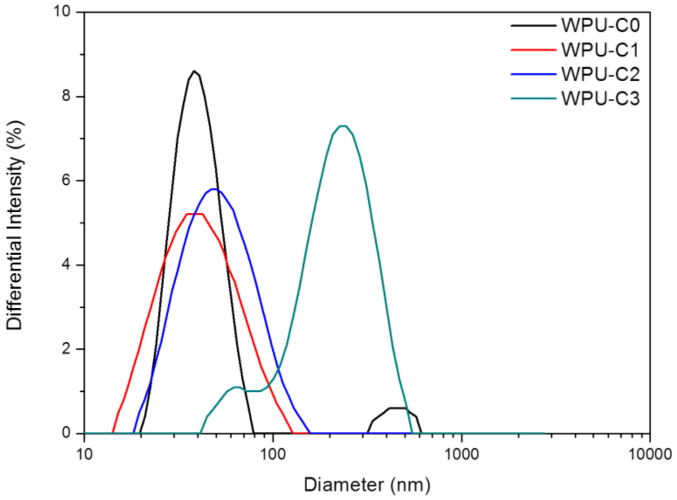

Figure S3. Particle size distribution of WPU emulsion.

Table S1. Number average particle size of WPU emulsions.

Sample code	Number average diameter (nm)
WPU-C0	40.8
WPU-C1	37.1
WPU-C2	48.3
WPU-C3	182.5

Table S2. Gel contents of WPU films.

Sample code	Gel content* (%)
WPU-C0	0.35 (±0.03)
WPU-C1	48.85 (±0.71)
WPU-C2	61.58 (±0.24)
WPU-C3	66.53 (±4.37)

* Determined from the ratio of sample weight after removal of soluble parts and the weight of pristine WPU film before the dissolution in THF/DMF mixture (50/50 by wt) at room temperature.

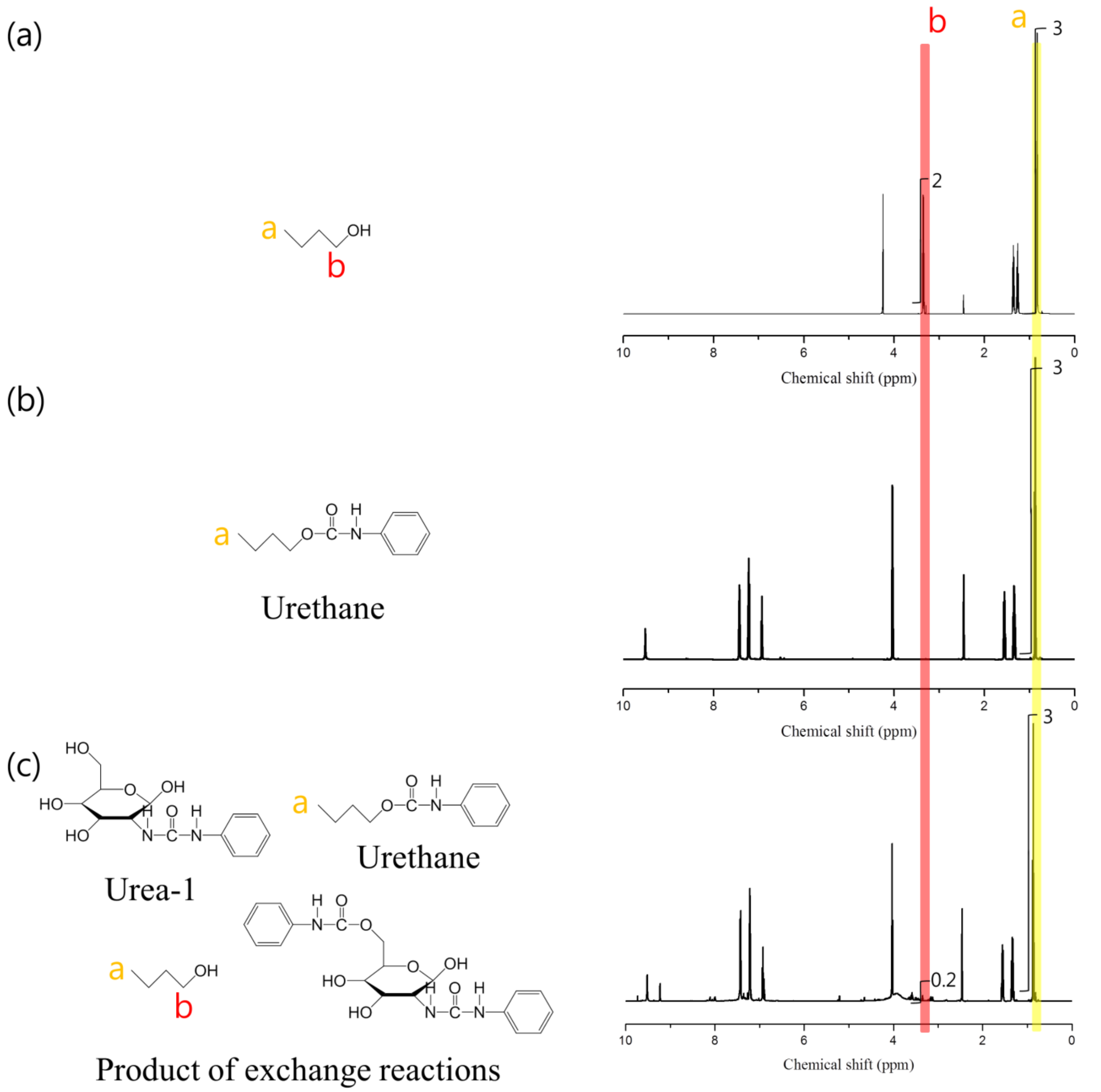

Figure S4. ^{1}H NMR spectra of the model compounds for the exchange reaction between Urea-1 and Urethane: (**a**) butanol; (**b**) Urethane; (**c**) products of the exchange reactions between Urea-1 and Urethane.

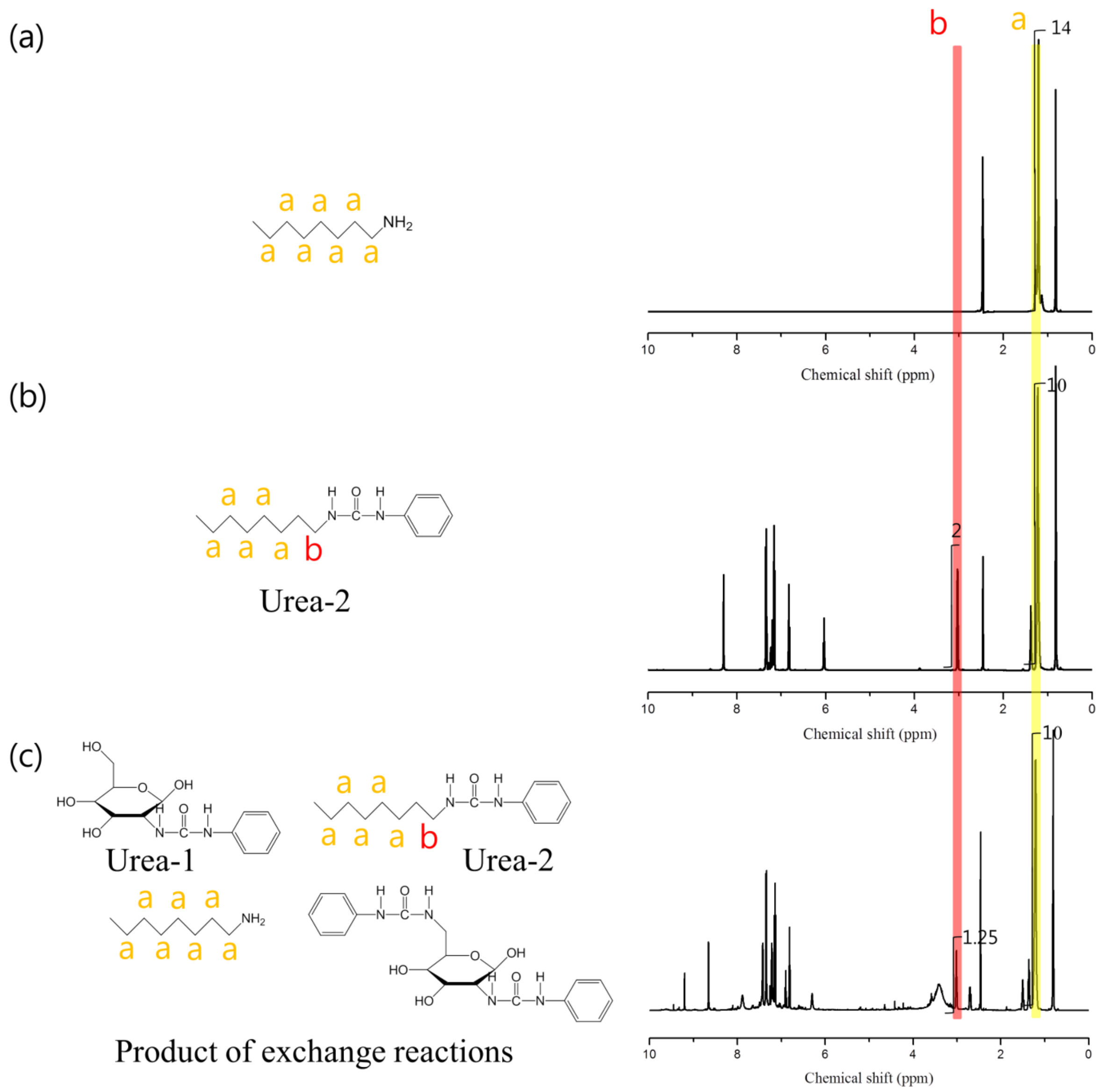

Figure S5. ^{1}H NMR spectra of the model compounds for the exchange reaction between Urea-1 and Urea-2: (**a**) octyl amine; (**b**) Urea-2; (**c**) products of the exchange reactions between Urea-1 and Urea-2.

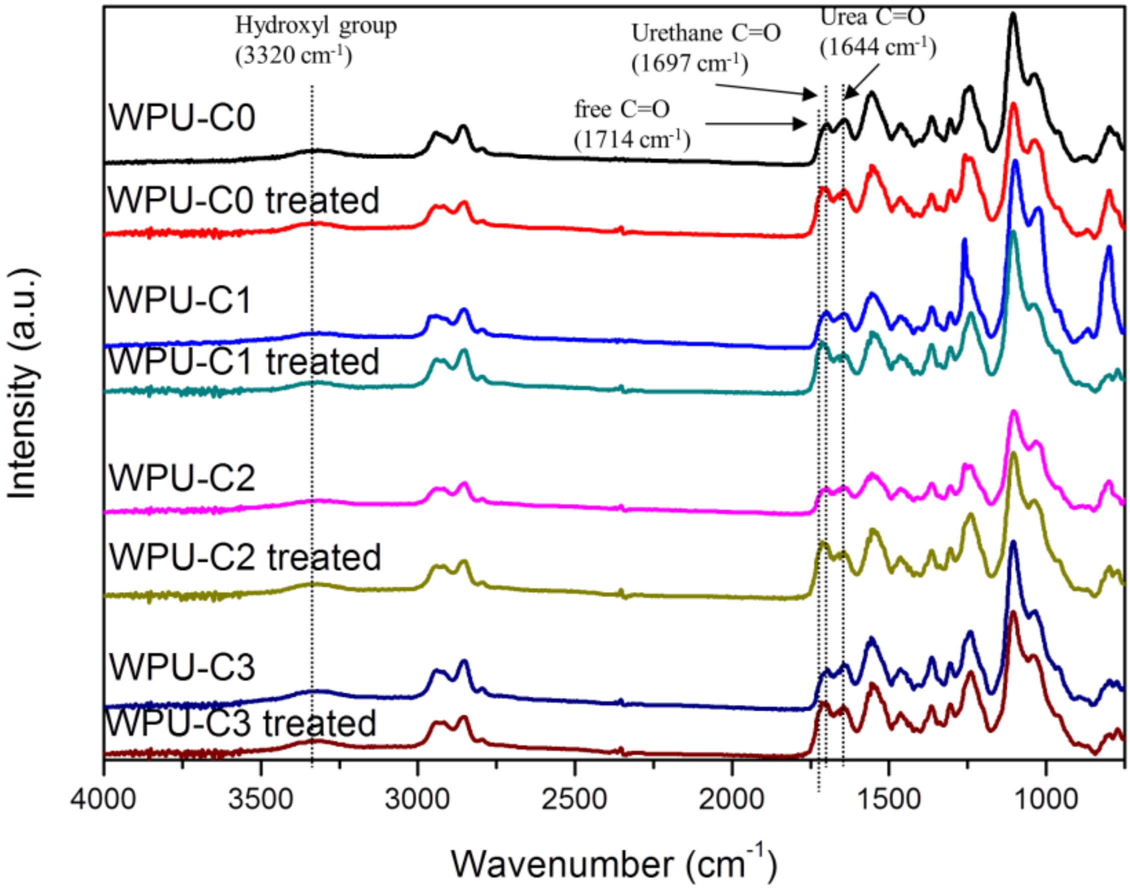

Figure S6. The FT-IR spectra of polyurethanes (PU) films before and after heat treatment for 24 h at 110 °C.

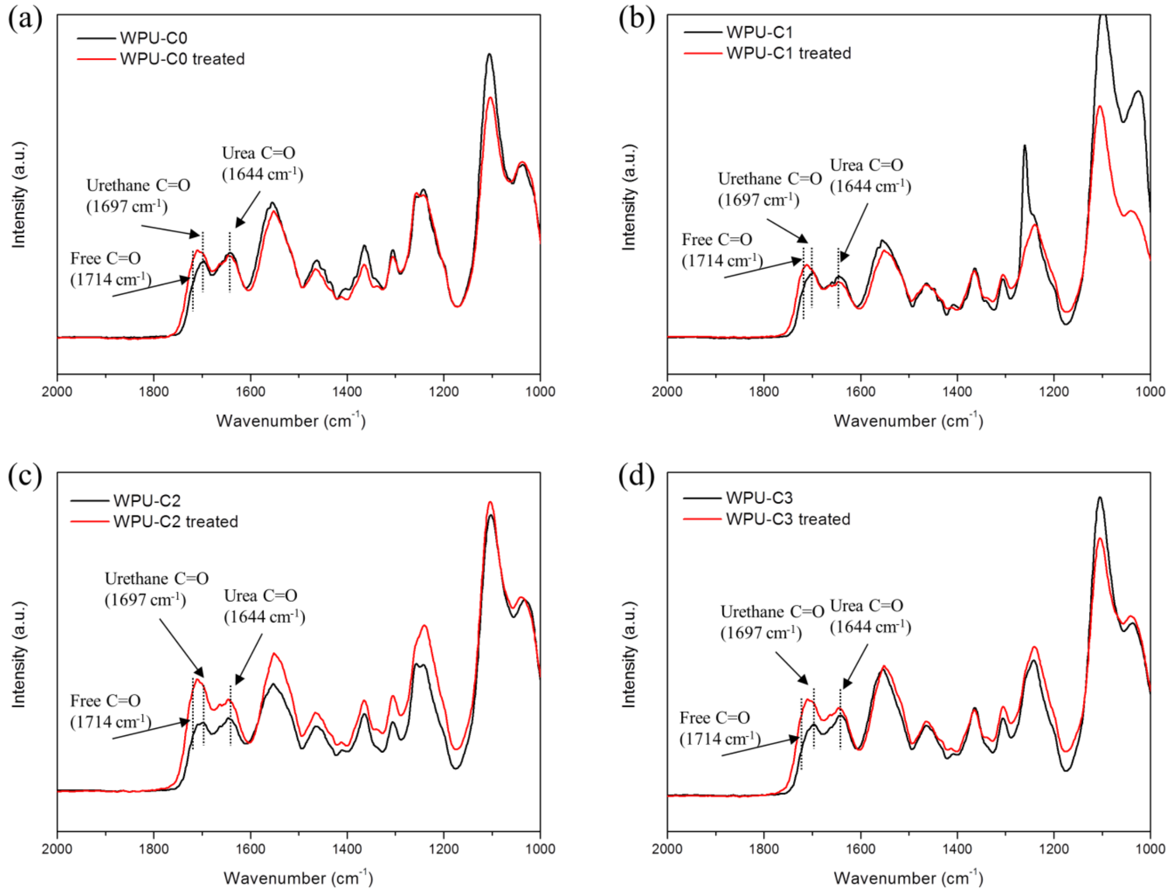

Figure S7. FT-IR spectra of WPU films before and after heat treatment for 24 h at 110 °C in the wavenumber of 2000~1000 cm^{-1}.

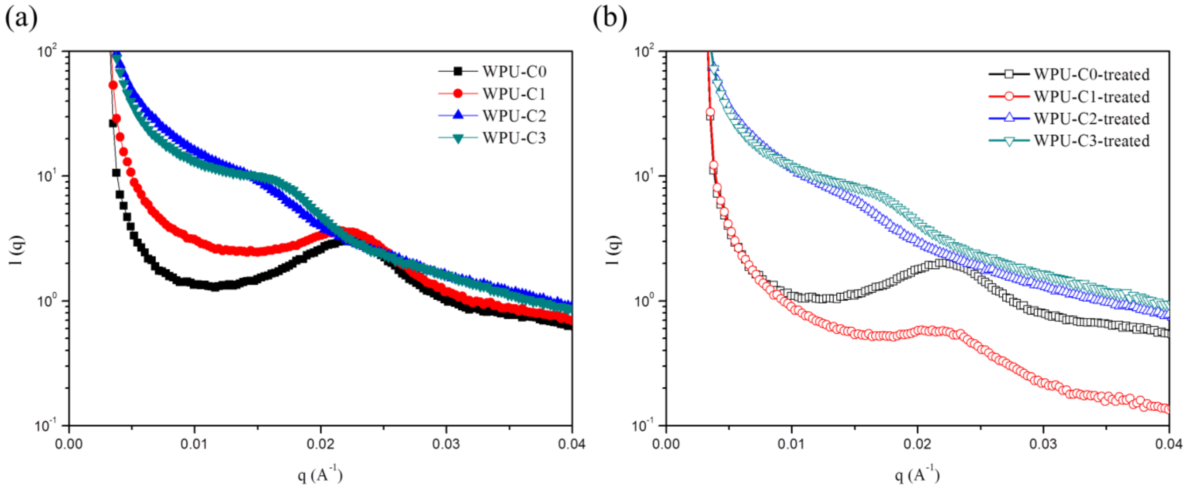

Figure S8. SAXS data of WPU films before and after heat treatment for 24 h at 110 °C.

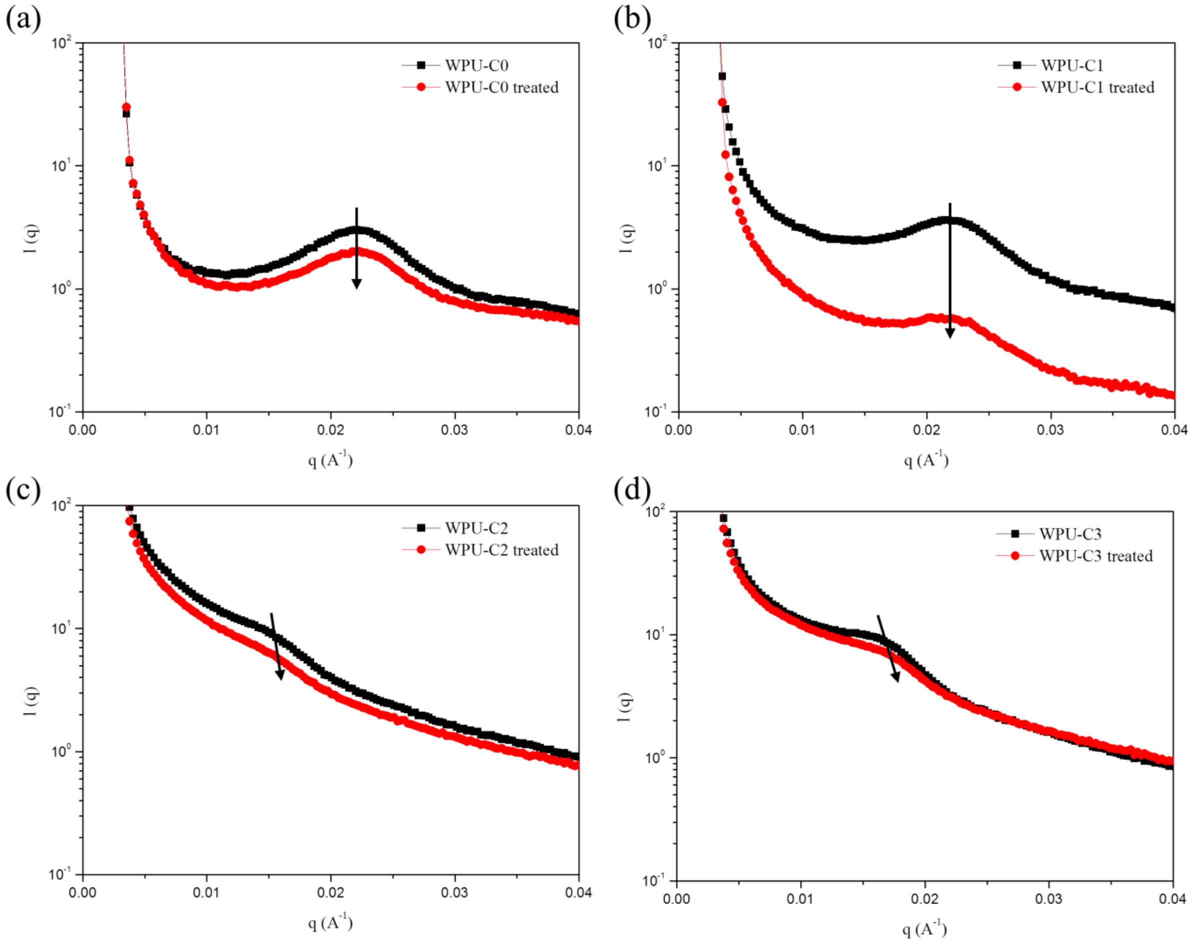

Figure S9. SAXS data of WPU films before and after heat treatment for 24 h at 110 °C.

Table S3. d-spacing of WPU films based on SAXS data before and after heat treatment for 24 h at 110 °C.

Sample code	d-spacing (nm)	
	Before heat treatment	After heat treatment
WPU-C0	28	28
WPU-C1	29	29
WPU-C2	35	33
WPU-C3	38	35

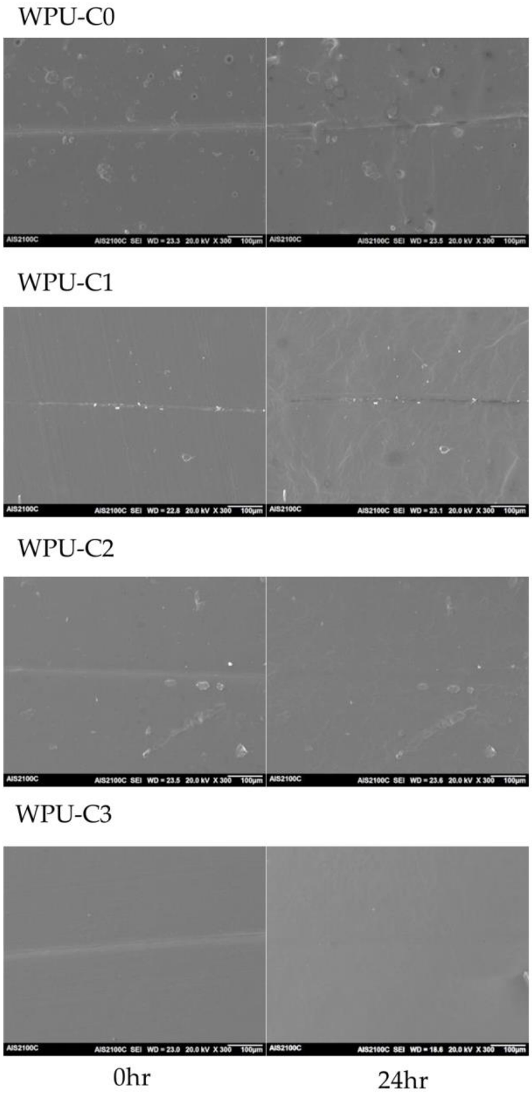

Figure S10. SEM images of the WPU films after the second self-healing test by the additional scratch and heat treatments at 110 °C for 24 h.
